# Molecular, neuromuscular, and recovery responses to light versus heavy resistance exercise in young men

**DOI:** 10.14814/phy2.13457

**Published:** 2017-09-28

**Authors:** Cody T. Haun, Petey W. Mumford, Paul A. Roberson, Matthew A. Romero, Christopher B. Mobley, Wesley C. Kephart, Richard G. Anderson, Ryan J. Colquhoun, Tyler W. D. Muddle, Michael J. Luera, Cameron S. Mackey, David D. Pascoe, Kaelin C. Young, Jeffrey S. Martin, Jason M. DeFreitas, Nathaniel D. M. Jenkins, Michael D. Roberts

**Affiliations:** ^1^ School of Kinesiology Auburn University Auburn Alabama; ^2^ Applied Neuromuscular Physiology Laboratory Oklahoma State University Stillwater; ^3^ Department of Cell Biology and Physiology Edward Via College of Osteopathic Medicine – Auburn Campus Auburn Alabama

**Keywords:** Electromyography, mammalian target of rapamycin, postexercise recovery, resistance training

## Abstract

Recent evidence suggests that resistance training with light or heavy loads to failure results in similar adaptations. Herein, we compared how both training modalities affect the molecular, neuromuscular, and recovery responses following exercise. Resistance‐trained males (mean ± SE: 22 ± 2 years, 84.8 ± 9.0 kg, 1.79 ± 0.06 m; *n* = 15) performed a crossover design of four sets of leg extensor exercise at 30% (light RE) or 80% (heavy RE) one repetition maximum (1RM) to repetition failure, and heavy RE or light RE 1 week later. Surface electromyography (EMG) was monitored during exercise, and vastus lateralis muscle biopsies were collected at baseline (PRE), 15 min (15mPOST), and 90 min following RE (90mPOST) for examination of molecular targets and fiber typing. Isokinetic dynamometry was also performed before (PRE), immediately after (POST), and 48 h after (48hPOST) exercise. Dependent variables were analyzed using repeated measures ANOVAs and significance was set at *P* ≤ 0.05. Repetitions completed were greater during light RE (*P* < 0.01), while EMG amplitude was greater during heavy RE (*P* ≤ 0.01). POST isokinetic torque was reduced following light versus heavy RE (*P* < 0.05). Postexercise expression of mRNAs and phosphoproteins associated with muscle hypertrophy were similar between load conditions. Additionally, p70s6k (Thr389) phosphorylation and fast‐twitch fiber proportion exhibited a strong relationship after both light and heavy RE (*r* > 0.5). While similar mRNA and phosphoprotein responses to both modalities occurred, we posit that heavy RE is a more time‐efficient training method given the differences in total repetitions completed, lower EMG amplitude during light RE, and impaired recovery response after light RE.

## Introduction

A recent scientific debate has centered around whether training with relatively light versus heavy resistance training (RT) loads to repetition failure similarly increases skeletal muscle hypertrophy and strength (Burd et al. 2010, 2013; Mitchell et al. 2012; Schuenke et al. 2013; Jenkins et al. 2016a; Morton et al. 2016b). Indeed, recent studies comparing acute and subchronic responses to RT have revealed strikingly similar anabolic signaling outcomes (Creer et al. 2005; Dreyer et al. 2006; Burd et al. 2010; Fry et al. 2010; Mitchell et al. 2012; Morton et al. 2016b). Recent reports have also indicated that heavy and light loads yield similar strength‐related outcomes (Morton et al. 2016a; Jenkins et al. 2017). In lieu of these findings, recent scientific debates have ensued questioning whether light and heavy resistance training similarly facilitate hypertrophic and strength adaptations (Burd et al. 2013; Schuenke et al. 2013).

With regard to the acute molecular response to light versus heavy resistance exercise, Burd et al. (2010) recently reported that the acute anabolic response to light (30% 1RM) unilateral knee extension RE to failure resulted in significantly greater myofibrillar fractional protein synthesis rates than heavy RE to failure (90% 1RM) 24 h postexercise, and that this response was potentially mediated through greater mechanistic target of rapamycin complex 1 (mTORC1) signaling. Briefly, the mTORC1 pathway is largely responsible for increases in myofibrillar protein synthesis (MPS) in response to one bout of resistance exercise (RE) (Baar and Esser 1999; Hornberger et al. 2004; Ogasawara et al. 2016). p70s6k is a downstream effector of mTORC1 signaling and past rodent and human studies have indicated that p70s6k phosphorylation magnitude in response to a single bout of RE may exhibit a predictive relationship of skeletal muscle hypertrophy over longer term periods (Baar and Esser 1999; Terzis et al. 2008). Notwithstanding, limited data exist describing how light versus heavy RE affects mTORC1 signaling intermediaries in skeletal muscle.

Acute RE dynamically affects mRNA expression patterns related to anabolism (e.g., a downregulation in myostatin [Dalbo et al. 2013], and an upregulation in genes related to satellite cell activation and ribosome biogenesis [Roberts et al. 2010; Figueiredo et al. 2016]), proteolysis (e.g., a downregulation in atrogenes [Dalbo et al. 2013]), and sarcomerogenesis (e.g., an upregulation in myosin heavy chain isoforms [Willoughby and Nelson 2002]). Moreover, acute alterations in select mRNA transcripts related to NF‐*κ*B and TNF‐*α* signaling after one bout of RE have been shown to correlate to hypertrophic outcomes following long‐term RT (Raue et al. 2012). A limited number of investigations have examined the mRNA response of genes related to satellite cell physiology (e.g., Pax7, MyoD, and myogenin [Burd et al. 2010]) and sarcomerogenesis (e.g., myosin heavy chain isoforms [Schwarz et al. 2015]) in response to light and heavy RE, albeit little evidence beyond these reports have examined acute postexercise mRNA expression patterns in response to light and heavy RE.

Beyond the molecular responses that occur in response to RE, the functional recovery response to RE is also critical to consider. Indeed, RE increases postexercise muscle soreness and/or impairs force production recovery for up to 2–7 days following RE (Byrne et al. 2004). However, to our knowledge, no studies have examined how light versus heavy RE to repetition failure comparatively affects torque recovery and soreness for up to 48 h after RE within the same subjects.

Therefore, the purpose of this study was to examine the molecular, neuromuscular, and functional postexercise recovery responses to light (i.e., 30% one repetition maximum [1RM]) versus heavy (i.e., 80% 1RM) RE to concentric repetition failure in young men. Specifically, training for the light and heavy sessions involved four sets of leg extensor exercise, molecular analyses were performed on vastus lateralis muscle biopsy samples, and neuromuscular analyses were performed on a region of the thigh in close proximity to the muscle sampling sites. With intent for parsimonious statistical inference and removal of any bias, we assumed the null hypothesis as true; namely, an assumption that no significant differences would exist between any dependent variable based on loading condition and/or the influence of fiber‐type proportion.

## Materials and Methods

### Ethical approval

Prior to initiating this study, the protocol was reviewed and approved by the Auburn University Institutional Review Ethics Committee, and was in compliance with the Helsinki Declaration. Subjects gave written consent and completed a Physical Activity Readiness Questionnaire and a health history questionnaire to detect potential risk factors that might be aggravated by strenuous physical activity. Healthy resistance‐trained young men were recruited to take part in this investigation. All participants confirmed that they had been completing ≥3 days per week of RE for ≥6 months with at least one, weekly lower body RE session.

### Experimental design

Fifteen subjects (age: 22 ± 2 years old, body mass: 84.8 ± 9.0 kg, body mass index: 26.4 ± 2.0 kg/m^2^, leg extensor 1RM: 120 ± 28 kg) participated in the current investigation. Subjects visited the laboratory on five separate occasions for data collection (T1–T5). A description of each visit and the experimental tests for each visit are provided below. Subjects were instructed to arrive at the same time of day for all visits in a hydrated state and at least 4 h fasted.

#### Visit 1 (T1)

Upon completion of paperwork, anthropometric measurements of height and body mass were completed using a stadiometer (seca769, CA). After this, subjects' 1RM was measured on a bilateral leg extension machine. The administered 1RM test protocol required subjects to lift the weight to a fully extended position from a starting position (knees bent at ~90°). A metronome beeping at 40 beats per minute was employed to ensure a controlled repetition and consistency among subjects. Load sequencing for the 1RM test consisted of subjects beginning the testing protocol with a warm‐up set of 10 repetitions with 20.4 kg (45 lbs). After the warm‐up set and between all sets of the 1RM protocol, subjects were given a minimum of 1 min, but up to 2 min of rest. After the warm‐up set, sets of one repetition were executed. 11.3 kg (25 lbs) was added to the machine after each successful set until the subject was unable to fully extend their legs and hold the position for the requisite time between metronome beeps. Upon establishing 1RM, subjects were randomly assigned to either the 30% 1RM (light) or 80% 1RM (heavy) load for T2 and T4 visits described below.

#### T2–T5 visits

**Figure 1:**
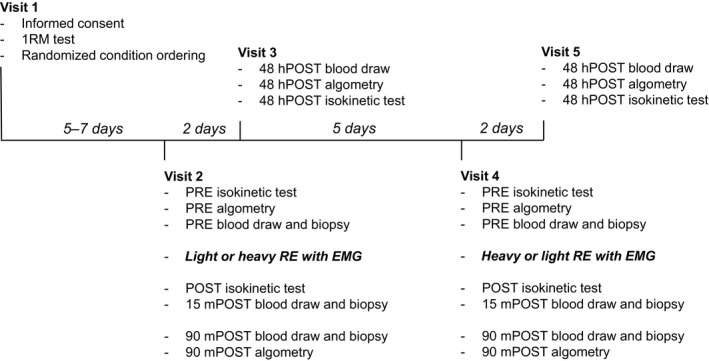
Study design. Data collection procedures for each visit and the time between visits.

T2 was scheduled 1 week after T1, T3 occurred 48 h thereafter, T4 occurred 1 week after T2, and T5 occurred 48 h after T4 (see Fig. 1 below for experimental timeline). Subjects were instructed to abstain from intense lower‐body exercise within 48 h of Visits 1, 2, and 4. Additionally, subjects were asked to refrain from intense upper‐body exercise within 24 h of all visits.

#### T2 and T4 visits (30% or 80% lifting bouts)

Upon arrival to the laboratory for T2 and T4, subjects' baseline pressure‐to‐pain threshold (PPT) of the right vastus lateralis was measured using a handheld algometer (Force Ten FDX, Wagner Instruments, Greenwich, CT). Briefly, focal pressure was applied by the algometer to visually sectioned proximal, medial, and distal portions of the left vastus lateralis which were marked for accurate application of force. Algometry pressure was applied at a rate of approximately 5 Newtons per second (N•s^−1^) at each site until the subject audibly indicated the specific moment at which the applied pressure became painful. At this point, the PPT value in N was recorded. The digital display of the algometer indicating the force value was blinded to the participants. The PPT was measured sequentially from proximal, medial, and distal sites, respectively, three times for triplicate measures with ~30 sec between cycles of measurement. The average of the triplicate measures at each site was calculated as the respective PPT of the site, and these values were averaged for a total PPT of the vastus lateralis. Algometry data were collected for all subjects at the PRE and POST time points (*n* = 15). However, algometry data were collected for only 11 subjects at the 48hPOST time points after T2 and T4 due to circumstantial absences of four subjects at these time points.

After PPT measurement, baseline venous blood sampled from the antecubital vein was collected into a 5‐mL serum separator tube (BD Vacutainer, Franklin Lakes, NJ) for select analysis of serum markers described below. After venipuncture, subjects completed isokinetic strength testing and were prepped for surface electromyography. After isokinetic dynamometry testing, subjects were instructed to lay in a supine position on a treatment table whereby a baseline (PRE) percutaneous skeletal muscle biopsy was obtained from the right vastus lateralis midway between the patella and iliac crest using a 5‐gauge needle with suction and sterile laboratory procedures previously described by our laboratory (Martin et al. 2016). Upon biopsy sample collection, sample portions were separately processed for mRNA, protein, and immunohistochemical analyses. Approximately 75 mg of muscle tissue was placed in foil and snap‐frozen in liquid nitrogen for eventual batch‐processing. Samples in foil were placed in a ‐80°C freezer, and upon removal, were processed according to the procedures described below. Approximately 50 mg of tissue was placed in a 1.7 mL polypropylene tube containing 500 ?L of nondenaturing cell lysis buffer (Cell Signaling, Danvers, MA) with preadded protease and phosphatase inhibitors and processed for protein analyses described later described later in the methods. Additionally, 10–20 mg of muscle was placed in a 1.7‐mL polypropylene tube containing 500 *μ*L of Ribozol (Ameresco, OH) for mRNA analyses described below. The remaining tissue (~100 mg) was placed in a small plastic tissue molder, slow‐frozen in optimal cutting temperature (OCT) media in liquid nitrogen‐cooled isopentane, and stored at −80°C for immunohistochemistry analyses described below. Cell lysate supernatants and tissue placed in Ribozol were also frozen at −80°C until batch processing described later. Any remaining tissue was snap‐frozen in liquid nitrogen and subsequently stored at −80°C. After 45 min of passive rest, subjects completed four sets of as many technically proficient repetitions as possible of bilateral leg extensions with either 30% or 80% of their tested 1RM. An unsuccessful repetition, characterized by incomplete extension of the legs at the requisite tempo of the metronome, resulted in termination of the set. Subjects were given 3 min of rest between sets. Immediately after the bout of RE (POST), isokinetic dynamometry measurements were performed which lasted ~15 min, and a second venipuncture and muscle biopsy were performed thereafter (15mPOST). Subjects then passively rested for ~90 min before a second assessment of soreness via algometry occurred, followed by a third blood draw and third muscle biopsy (90mPOST).

#### T3 and T5 visits (48 h postbout recovery assessment)

Upon arrival to the laboratory for T3 or T5, subjects were first assessed for appropriate recovery of biopsy sites. Thereafter, algometry measurements were collected, a venipuncture was completed to assess serum markers of muscle damage, and isokinetic dynamometry measurements were collected to assess force production parameters using the methods described for T2/4 visits.

### Serum myoglobin analyses

As mentioned previously, blood was collected at baseline (PRE), 15 min postexercise (15mPOST), and 48 h postexercise (48hPOST). Upon collection in serum tubes, blood was immediately centrifuged at 4000*g* for 5 min at 4°C. Serum was then separated into cryotubes and immediately placed in a −80°C freezer for later batch processing of myoglobin. Myoglobin concentration in serum samples was determined using a commercially available immunoperoxidase assay (GWB‐AF81CB; Genway Biotech, San Diego, CA). Serum myoglobin data were collected from only 11 subjects at the 48hPOST time points due to circumstantial absence of four subjects. The mean intra‐assay CV for all duplicates was 5.3%.

### Muscle mRNA expression analyses

Following muscle extraction, samples were homogenized in 500 *μ*L of Ribozol (Ameresco) and stored at −80°C for batch processing as described above. During batch processing, total RNA isolation occurred according to manufacturer's instructions. RNA concentrations were subsequently assessed using a NanoDrop Lite (Thermo Fisher Scientific, Waltham, MA) prior to cDNA synthesis. cDNA synthesis was reverse transcribed from 1000 ng of total RNA for real‐time PCR analyses using a commercial cDNA synthesis kit (Quanta Biosciences, Gaithersburg, MD). Real‐time PCR was performed using SYBR‐green‐based methods with gene‐specific primers designed using an online primer designer tool (Primer3Plus, Cambridge, MA) (genes are denoted in Table 1). The comparative threshold cycle (CT) method (2^−ΔΔCT^) was employed to calculate relative gene expression (Schmittgen and Livak 2008). Fold‐change values at 15mPOST and 90mPOST time points were calculated by normalizing CT values to baseline gene expression. Melt curve analyses were performed on the first PCR plate for each GOI to ensure that one PCR product was amplified per reaction. Due to a lack of sufficient muscle tissue availability from 2 subjects for RNA isolation techniques, only 13 subject's data were used for mRNA expression analysis.

**Table 1:** Primer sequences for real‐time PCR

Gene	Forward primer (5′ → 3′)	Reverse primer (5′ → 3′)
Hypertrophy and proteolysis		
MGF	CGAAGTCTCAGAGAAGGAAAGG	ACAGGTAACTCGTGCAGAGC
MSTN	GACCAGGAGAAGATGGGCTGAATCCGTT	CTCATCACAGTCAAGACCAAAATCCCTT
MURF‐1	GCCTTCTTCGCCTTCTCC	AGCTCATACAGACTCAGTTCC
Atrogin‐1	ATGTGCGTGTATCGGATGG	AAGGCAGGTCAGTGAAGC
Mitochondrial biogenesis		
PGC1‐*α*	CAAGCCAAACCAACAACTTTATCTCT	CACACTTAAGGTGCGTTCAATAGTC
Inflammation		
IL‐6	AGGAGACTTGCCTGGTGAAA	CAGGGGTGGTTATTGCATCT
TNF‐*α*	TCCTTCAGACACCCTCAACC	AGGCCCCAGTTTGAATTCTT
Sarcomere scaffolding proteins		
Nebulin	GAAGCCAACAAAGCCCACTG	AAAATCGCTTTGCTGCAGGG
Titin	CCGAAATGCATCAGTCAGCG	CTGTAGCTGAACACTGGCCA
Myosin heavy chain isoforms		
MHC I	GTATGAGGAGTCGCAGTCGG	AGGGACTCCTCATAGGCGTT
MHC IIa	GAACACCCAAGGCATCCTCA	GCTGTTCCTTCAGGTCCTCC
MHC IIx	CTGGTGGACAAACTGCAAGC	CCTGCGGAATTTGGAGAGGT
Housekeeping gene		
Fbl	CCCACACCTTCCTGCGTAAT	GCTGAGGCTGTGGAGTCAAT

All primers were designed using PrimerPlus3 (Cambridge, MA) and BLASTed against other potential mRNA targets using the online NCBI Nucleotide database (Bethesda, MD).

### Western blotting

Following muscle extraction, samples were homogenized using a tight‐fitting micropestle in cell lysis buffer as described above. Insoluble proteins were removed by centrifugation at 500*g* for 5 min at 4°C, and supernatants containing muscle tissue homogenate were collected and stored at −80°C. After all participants finished the study, muscle tissue homogenates were batch assayed for total protein content using a BCA Protein Assay Kit (Thermo Fisher Scientific, Waltham, MA). Cell lysis homogenates obtained from above were prepared for western blotting using 4× Laemmli buffer at 1 *μ*g/*μ*L. Thereafter, 25 *μ*L of prepped samples were loaded onto 4–15% SDS‐polyacrylamide gels (BioRad, Hercules, CA) and subjected to electrophoresis (180 V for 60 min) using 1× SDS PAGE running buffer (Ameresco). Proteins were then transferred to polyvinylidene difluoride membranes (BioRad), Ponceau stained, and imaged using a gel documentation system (UVP, Upland, CA) to ensure equal protein loading between lanes and to normalize phosphoprotein expression values described below. Thereafter, membranes were blocked for 1 h at room temperature with 5% nonfat milk powder. Rabbit antiphosphorylated AMPK (Thr172) (1:1000; Cell Signaling), rabbit antiphosphorylated mTOR (Ser2448) (1:1000; Cell Signaling), rabbit antiphosphorylated p70s6k (Thr389) (1:1000; Cell Signaling), rabbit antiphosphorylated rps6 (Ser235/236) (1:1000; Cell Signaling), and rabbit antiphosphorylated 4EBP1 (Thr37/46) (1:1000, Cell Signaling) were incubated with membranes overnight at 4°C in 5% bovine serum albumin (BSA). The following day, membranes were incubated with horseradish peroxidase‐conjugated anti‐rabbit IgG (1:2000, Cell Signaling) at room temperature for 1 h prior to membrane development. Membrane development was performed using an enhanced chemiluminescent reagent (Luminata Forte HRP substrate; Millipore, Billerica, MA), and band densitometry was performed through the use of a gel documentation system and associated densitometry software (UVP). Of note, the densitometry values for all protein targets were normalized to Ponceau densities, and these values were normalized to PRE values in order to obtain fold‐change values where: POST/PRE = fold change at 15mPOST and 90mPOST/PRE = fold change at 90mPOST. Due to a lack of sufficient muscle tissue availability from 2 subjects for protein isolation techniques, only 13 subject's data were used for protein expression analysis.

### Immunohistochemistry methods

**Figure 2:**
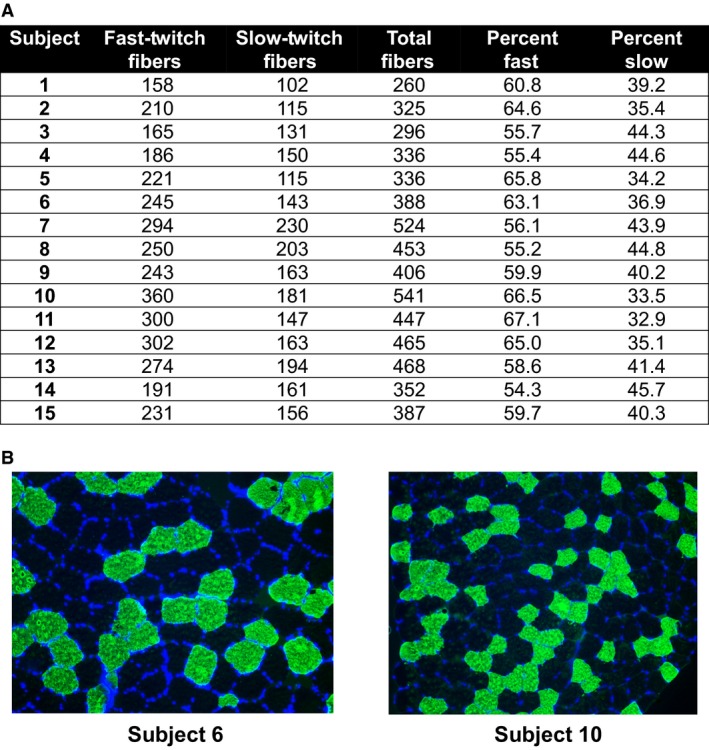
Subject fiber‐type data. (A) The number and percentage of fast‐ and slow‐twitch muscle fibers of each subject. (B) Two representative 20× objective images of subject 6 and subject 10.

Methods employed previously in our laboratory and described elsewhere were used for the immunohistochemistry technique in this investigation (Hyatt et al. 2015; Martin et al. 2016). Sections from OCT‐preserved samples were cut at a thickness of 20 *μ*m using a cryotome (HM 525 Cryostat; Thermo Fisher Scientific, Waltham, MA) and were adhered to positively charged histology slides. Once all samples were sectioned, batch processing occurred for immunohistochemistry. Briefly, sections were dried at room temperature for 30 min and incubated in a phosphate‐buffered saline (PBS) solution containing 0.5% Triton X‐100, and blocked with Pierce Super Blocker (Thermo Fisher Scientific). Thereafter, sections were rinsed in PBS and incubated with primary antibodies for 1 h. The primary antibodies used for muscle fiber typing were MHC type I (mouse IgG isotype, # A4.951; Developmental Studies Hybridoma Bank) at a 1:15 dilution and mouse IgM anti‐sarcoglycan (Thermo Fisher Scientific). Primary antibodies were diluted in PBS containing 0.5% Pierce Super Blocker (Thermo Fisher Scientific). After primary antibody incubation, slides were washed in PBS and incubated at a 1:100 dilution of Alexa Fluor 488 goat anti‐mouse IgG and Alexa Fluor 350 goat anti‐mouse IgM secondary antibodies (Molecular Probes) diluted in PBS containing 0.5% Pierce Super Blocker (Thermo Fisher Scientific). After secondary antibody incubation, sections were washed in PBS and mounted using DAPI‐containing fluorescent mounting media (Burlingame, CA) and imaged with a fluorescence microscope (Nikon Instruments, Melville, NY). Importantly, muscle fiber typing utilizing this method allows for the individual visualization of the muscle fiber membrane, myonuclei using the DAPI filter set (blue), and type I (using the FITC filter set [green]) and type II muscle fibers (nonstained/black). For each time point, three 10× objective images of each respective fluorescent filter were obtained using a fluorescent microscope (Nikon Eclipse Ti‐U; Nikon Instruments, Melville, NY). Images were merged using the NIS Elements software (Nikon). Slides for each subject from each time point contained ~75–100 fibers. Thus, given the availability of ~600 total fibers from each subject from all time points combined, ~400 fibers found on slides from which clear images were available were used to phenotype each subject's percent fast and percent slow fibers. CellProfiler™ open‐sourced software (Carpenter et al. 2006) was used to objectively quantify the amount of fast and slow fibers per image by converting images to grayscale and counting the number of slow and fast fibers. With this method, green fibers (MHC‐I) were converted to light gray or mostly white, and black fibers (MHC‐IIa/x) remained black (see representative images in Fig. 2).

### Isokinetic dynamometry methods

For isokinetic testing, participants were seated with straps securing their trunk and pelvis on a calibrated isokinetic dynamometer (Biodex System 4, Biodex Medical Systems, Inc., Shirley, NY). The input axis of the dynamometer was aligned with the lateral epicondyle of the left femur and the seat was set so that the hip angle was approximately 90°. The range of motion for the isokinetic leg extension contractions was set from 5° to 95°, with 0° representing full leg extension. Prior to isokinetic testing, each participant performed a warm up consisting of submaximal to maximal isometric muscle actions. Following the isometric muscle actions, subjects completed two maximal voluntary isokinetic knee extension actions at 1.05 rad•s^−1^ (60°•s^−1^), 3.14 rad•s^−1^ (180°•s^−1^), and 5.24 rad•s^−1^ (300°•s^−1^) with approximately 30 sec of rest allowed between each contraction. The order of the isokinetic testing velocities was randomized at each visit and the left leg was used for all testing. Participants were provided verbal encouragement during each contraction, and concentric‐only leg extensor testing was performed. The isokinetic contractions resulting in the greatest peak value at each velocity were used for analyses. Isokinetic peak torque (PT; N·m) at 60°•s^−1^, 180°•s^−1^, and 300°•s^−1^ was calculated as the highest value that occurred during the isokinetic load range.

### Surface electromyography

A parallel bar, bipolar, surface electromyographic sensor (Delsys Trigno™, Delsys, Inc., Natick, MA) was placed on the vastus lateralis (VL) muscle of the left thigh. The contact material of the sensor was 99.9% silver, the interelectrode distance was 10 mm, the common mode rejection ratio was >80 dB, and the sampling rate was 1926 samples•s^−1^. The center of the sensor was placed at 66% of the distance between the anterior superior iliac spine (ASIS) and the lateral superior border of the patella. The sensor was oriented so that the longitudinal axis of the sensor was parallel to the angle of pennation of the vastus lateralis fibers (approximately 20°) (Fukunaga et al. 1997). To reduce interelectrode impedance and increase the signal‐to‐noise ratio, local areas of the skin were shaved, abraded with an abrasion pad, and cleaned with isopropyl alcohol prior to the placement of the electrodes (Beck and Housh 2008). An electrogoniometer was placed across the knee joint to monitor joint angle.

The EMG and electrogoniometer signals were sampled simultaneously and stored on a mobile personal monitor (Trigno™ mobile). Following completion of the study, the signals were downloaded to a personal computer and processed offline with custom written software (Labview v. 16.0, National Instruments, TX). The EMG signals were digitally filtered with a zero‐phase shift, fourth‐order Butterworth filter using a band pass of 10–499 Hz. The electrogoniometer signal was low‐pass filtered with a zero‐phase shift fourth‐order Butterworth filter using a 15‐Hz cutoff. All subsequent analyses were performed on the filtered signals. The EMG values were calculated from the signal epochs during the concentric portion of each repetition based on the electrogoniometer signal. The time domain of the EMG signals was expressed as the root mean square (RMS) amplitude value (EMG_AMP_). The absolute EMG_AMP_ values were expressed in *μ*V. The frequency domain of the EMG signals was expressed as the mean power frequency (MPF) in Hz. To quantify EMG_MPF_, each signal epoch was processed with a Hamming window and a discrete Fourier transformation (DFT) based on the recommendations of Diemont et al. (1988)) and calculated as described by Kwatny et al. (1970). The EMG_AMP_ and EMG_MPF_ values from the second repetition (denoted as initial repetition), a repetition corresponding to the middle of the completed set (middle repetition), and the last repetition were used for subsequent analyses (Jenkins et al. 2015). In addition, total integrated EMG amplitude (iEMG; *μ*V•s^−1^) was calculated as the sum of the total area under the rectified EMG signal during each set to quantify the total volume of muscle activation during each exercise bout. The values were normalized to the maximal corresponding EMG values during a maximal voluntary isometric contraction performed on an isokinetic dynamometer prior to the completion of the exercise bout when the subjects were unfatigued. Due to an issue with goniometer signal quality or the recording device, only the EMG data from 11 participants were used to examine muscle activation during exercise.

### Statistics

All statistics were performed using SPSS v23.0 (Chicago, IL). The assumption of normality of the residuals for all dependent variables was formally tested using the Shapiro–Wilks test. For normally distributed dependent variables, two‐way (Load [30% vs. 80% 1RM]) × (Time [level of time for each variable]) mixed‐factorial, repeated measures analyses of variance (ANOVAs) were performed. For any non‐normally distributed dependent variables, a square root transformation was performed and repeated measures ANOVAs were conducted on the transformed data. The homogeneity of variance assumption and Mauchly's tests of sphericity were also conducted for all dependent variables analyzed by ANOVA with more than two levels of time. In the event that sphericity was violated, the Huynh–Feldt correction was applied to the degrees of freedom and *P*‐value of the *F*‐statistic. If homogeneity of variance was violated, the Greenhouse–Geisser correction was applied to the *P*‐value. In the event that a significant load × time interaction occurred, the effect was regressed by conducting dependent samples *t*‐tests at each level of time in order to parse out the time point during which a significantly different effect of load existed. For select molecular variables, significant main effects of time were analyzed using Bonferroni post hoc tests. Significance was set at *P* ≤ 0.05. Correlations were also performed to examine the relationship between fiber‐type percentages and select dependent variables. Correlation coefficients are characterized as moderate (*r* = 0.3–0.5), strong (*r* = 0.5–0.7), very strong (*r* = 0.7–0.9), and nearly perfect (*r* = 0.9–1.0)(Hopkins 2000). In two cases where correlation coefficients exceeded 0.4, simple regression was performed where the dependent measure was regressed based on fast‐twitch fiber percentages. A scatter plot with a line of best fit, *r* value, and *P*‐value is provided to demonstrate relationships.

## Results

### Participant characteristics and muscle fiber typing

Participants (*N* = 15) were 22 ± 2 years old with a BMI of 26.4 ± 2.0 kg/m^2^ (height: 1.79 ± 0.06 m, body mass: 84.8 ± 9.0 kg). 1RM at baseline was 120 ± 28 kg. The average number of fast‐twitch fibers counted for all participants was 242 ± 57 and the average number of slow‐twitch fibers counted was 157 ± 35. The average percentage of fast‐twitch fibers was 60.51 ± 4.57% and the average percentage of slow‐twitch fibers was 39.49 ± 4.57% (Fig. 2).

### Effect of load on volume load and total repetitions per set

**Figure 3:**
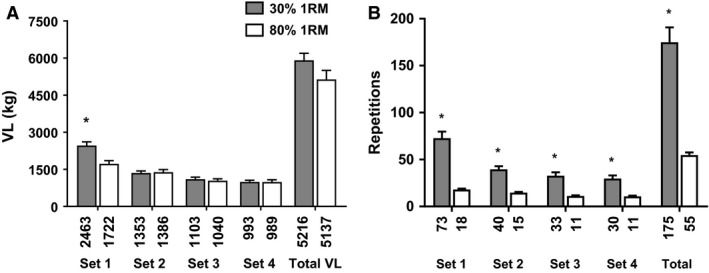
Volume load and repetition data. (A) Volume load (VL) differences between light and heavy load conditions. (B) Repetition number differences between light and heavy load conditions. All data are presented as mean ± SE (*n* = 15 per bar), and mean data are presented below each bar. * indicates a significant between‐condition difference (*P* < 0.05).

A significant main effect of time occurred for volume load (*F*_2,26_ = 72.10, *P *< 0.001) along with a significant load × time interaction (*F*_1.99,25.95_ = 14.95, *P *< 0.001; Fig. 3A). Dependent samples *t*‐tests revealed a significant difference in volume load between loads only for set 1 where the light condition volume load was 2463 ± 578 kg and the heavy condition volume load was 1721 ± 502 kg (742 ± 203 kg, *P *<* *0.001). A significant main effect of load (*F*_1,14_ = 66.02, *P *< 0.001), set (*F*_1.6,22.42_ = 68.39, *P *< 0.001), and load × set interaction occurred for repetitions per set (*F*_1.54,21.55_ = 37.77, *P *< 0.001; Fig. 3B). Dependent samples *t*‐tests revealed significant differences between all sets of 30% 1RM and 80% 1RM (p < 0.001) as well as a significant difference in the total number of reps between conditions (*P* < 0.001).

### Effect of load on electromyography

**Figure 4:**
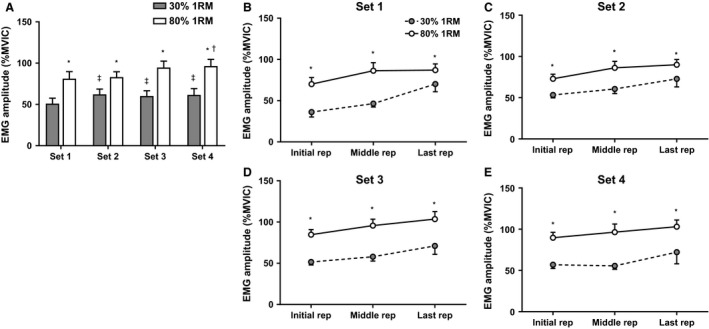
Electromyography data. (A) The difference between conditions in EMG amplitude means for each set. (B–E) The EMG amplitudes for each set (sets 1–4) expressed as a percentage of maximal voluntary isometric contraction during the initial rep, middle rep, and last rep of each set. All data are presented as mean ± SE (*n* = 11 per bar). * indicates a significant between‐condition difference (*P* < 0.05). † indicates set 4 > set 1 for 80% 1RM. ‡ indicates sets 2, 3, and 4 > set 1 for 30% 1RM.

For EMG amplitude, there was a main effect of repetition (*F*_2,18_ = 7.42, *P* = 0.02) and a load × set interaction (*F*_3,18_ **= 3.70, *P *= 0.02). During 30% 1RM exercise, EMG amplitude increased from set 1 to sets 2 (*P* = 0.02), 3 (*P* = 0.007), and 4 (*P* = 0.02) (Fig. 4A). During 80% 1RM exercise, EMG amplitude did not change from set 1 to set 2 (*P *>* *0.99) or 3 (*P* = 0.09), but increased to set 4 (*P* = 0.04) (Fig. 4A). EMG amplitude was greater during 80% 1RM than 30% 1RM exercise (*P* = 0.001–0.01) (Fig. 4B–E). Total iEMG was significantly greater during light than heavy RE (136.58 ± 25.48 vs. 61.83 ± 8.74; *P* = 0.001) (data not shown). For EMG_MPF_, a set × repetition interaction occurred (*F*_6,54_ = 2.33, *P* = 0.04) (data not shown). During set 1, EMG_MPF_ did not change from the first to middle repetition (*P *>* *0.99), but decreased during the last repetition (*P* = 0.01–0.02). However, there were no changes in EMG_MPF_ during sets 2 (*F*_1,10_ **= 1.65, *P* = 0.22), 3 (*F*_1,10_ **= 1.74, *P* = 0.20), or 4 (*F*_1,10_ = 1.00, *P* = 0.39). Furthermore, there was no change in EMG_MPF_ from sets 1 to 4 during the initial (*F*_1,10_ **= 2.47, *P* = 0.08) or middle repetition (*F*_1,10_ **= 1.88, *P* = 0.16). However, EMG_MPF_ decreased from set 1 to set 4 during the final repetition (*P* = 0.02).

### Effect of load on muscle soreness, serum myoglobin, and postexercise dynamometry measures

**Figure 5:**
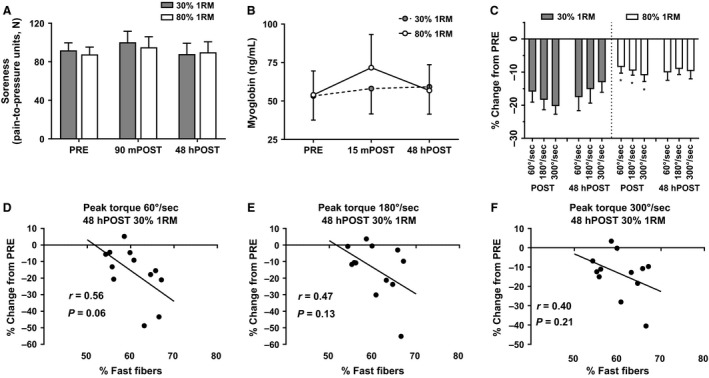
Soreness, myoglobin, and isokinetic dynamometry data. (A) The change in soreness measured by algometry. (B) The change in serum myoglobin. (C) The percent change in peak torque at each velocity at POST and 48hPOST each condition, relative to PRE. All data are presented as mean ± SE (*n* = 10 per bar for serum myoglobin and *n* = 11 per bar for isokinetic dynamometry). (D–F) The relationship between the percentage of fast‐twitch fibers and percent change in peak torque at each velocity 48hPOST light RE, relative to PRE. * indicates a significant between‐condition difference (*P* < 0.05).

A significant main effect of time (*F*_1,13_ = 7.77, *P* = 0.015) but not load (*F*_1,13_ = 0.77, *P* = 0.397) was revealed for average PPT (Fig. 5A). No significant load × time interaction occurred (*F*_1,13_ = 0.57, *P* = 0.463). In the subset of 11 subjects where data from all time points were available, a 2 × 3 repeated measures ANOVA revealed a significant load × time interaction (*F*_2,18_ = 4.65, *P* = 0.024). However, dependent samples *t*‐tests at each level of time revealed no significant differences in PPT between loads at any time point. A significant main effect of time (*F*_2,16_ = 5.14, *P* = 0.019) but not load (*F*_1,8_ = 1.37, *P* = 0.276) was revealed for serum myoglobin concentrations. No significant load × time interaction occurred (*F*_2,16_ = 2.17, *P* = 0.147) (Fig. 5B).

A significant main effect of time (*F*_1,13_ = 47.26, *P* = <0.001) and load (*F*_1,13_ = 7.32, *P* = 0.018) was discovered for average isokinetic peak torque (Fig. 5C). Furthermore, a significant load × time interaction (*F*_1,13_ = 10.48, *P* = 0.006) was discovered. A dependent samples *t*‐test revealed a significant difference between loads at POST only where average peak torque was 161.41 ± 31.10 N•m after lifting with light loads and 178.72 ± 29.88 N•m after lifting with heavy loads (mean difference: 17.31 ± 18.31 N•m, *P* = 0.003). Given these significant differences at POST, dependent samples *t*‐tests were completed comparing peak torques at each isokinetic velocity for each load at the POST time point. Significant differences in peak torque existed at all velocities whereby POST decrements in the light condition were greater than POST decrements in the heavy condition (*P* < 0.05).

At 48hPOST light RE, the following was observed: (1) a strong relationship approaching significance was discovered between fast fiber percentage and percent change in peak torque at 60°∙s^−1^ (*r* = −0.56, *P* = 0.060; Fig. 5E), and (2) nonsignificant associations existed between fast fiber percentage and percent change in peak torque at 180°∙s^−1^ (*r* = −0.47, *P* = 0.130; Fig. 5E) and peak torque at 300°∙s^−1^ (*r* = −0.40, *P* = 0.210; Fig. 5F).

### Skeletal muscle mRNA responses to both training loads

**Figure 6:**
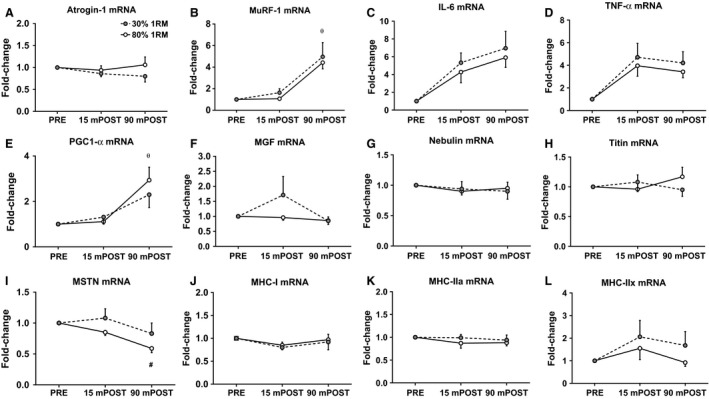
Muscle mRNA expression. (A–L) The fold change in each mRNA relative to PRE. All data are presented as mean ± SE (*n* = 13 per bar). *θ* indicates significant time effect whereby values were significantly greater than baseline regardless of load condition (*P* < 0.05); # indicates significant time effect whereby values were significantly less than baseline regardless of load condition (*P* < 0.05).

No significant effects of time (*F*_1,11_ = 0.026, *P* = 0.875), load (*F*_1,11_ = 0.21, *P* = 0.271), or load × time interaction (*F*_1,11_ = 0.43, *P* = 0.425) occurred for atrogin‐1 mRNA expression (Fig. 6A). A significant main effect of time (*F*_1,11_ = 21.17, *P* = 0.001), but no significant main effect of load (*F*_1,11_ = 0.26, *P* = 0.620) or a load × time interaction (*F*_1,11_ = 0.089, *P* = 0.771) occurred for MuRF‐1 mRNA expression (Fig. 6B). No significant effects of time (*F*_1,11_ = 2.24, *P* = 0.163), load (*F*_1,11_ = 2.3, *P* = 0.158), or load × time interaction (*F*_1,11_ = 0.01, *P* = 0.922) occurred for IL‐6 mRNA expression (Fig. 6C). No significant main effects of time (*F*_1,11_ = 0.26, *P* = 0.622), load (*F*_1,11_ = 0.77, *P* = 0.398), or load × time interaction (*F*_1,11_ = .002, *P* = 0.962) occurred for TNF‐*α* mRNA expression (Fig. 6D). A significant main effect of time (*F*_1,11_ = 9.91, *P* = 0.009), but no significant main effect of load (*F*_1,11_ = 0.01, *P* = 0.910) or load × time interaction (*F*_1,11_ = 0.89, *P* = 0.366) occurred for PGC1‐*α* mRNA expression (Fig. 6E). No significant effects of time (*F*_1,11_ = 1.87, *P* = 0.199), load (*F*_1,11_ = 0.91, *P* = 0.361), or load × time interaction (*F*_1,11_ = 2.20, *P* = 0.166) occurred for MGF mRNA expression (Fig. 6F). No significant effects of time (*F*_1,11_ = 0.03, *P* = 0.868), load (*F*_1,11_ = 0.05, *P* = 0.824), or load × time interaction (*F*_1,11_ = 0.04, *P* = 0.842) occurred for nebulin mRNA expression (Fig. 6G). No significant effects of time (*F*_1,11_ = 0.14, *P* = 0.721), load (*F*_1,11_ = 0.40, *P* = 0.539), or load × time interaction (*F*_1,11_ = 0.75, *P* = 0.405) occurred for titin mRNA expression (Fig. 6H). A significant effect of time (*F*_1,11_ = 5.52, *P* = 0.039), but no significant main effect of load (*F*_1,11_ = 2.07, *P* = 0.178), or load × time interaction (*F*_1,11_ = 0.02, *P* = 0.894) occurred for MSTN mRNA expression (Fig. 6I). No significant effects of time (*F*_1,11_ = 0.74, *P* = 0.409), load (*F*_1,11_ = 0.53, *P* = 0.480), or load × time interaction (*F*_1,11_ = 0.02, *P* = 0.893) occurred for MHC‐I gene expression (Fig. 6J). No significant effects of time (*F*_1,11_ = 0.45, *P* = 0.515), load (*F*_1,11_ = 0.88, *P* = 0.369), or load × time interaction (*F*_1,11_ = 0.21, *P =* 0.657) occurred for MHC‐IIa mRNA expression (Fig. 6K). No significant effects of time (*F*_1,11_ = 1.10, *P* = 0.317), load (*F*_1,11_ = 1.32, *P* = 0.275), or load × time interaction (*F*_1,11_ = 0.05, *P* = 0.826) occurred for MHC‐IIx mRNA expression (Fig. 6L).

### mTORC1 signaling responses to both training loads

**Figure 7:**
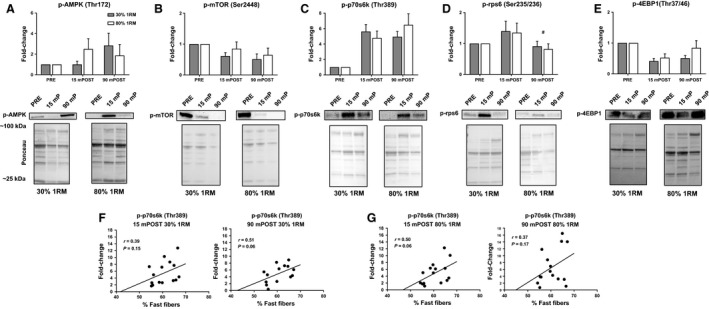
Muscle phosphoprotein expression. (A–E) The fold change in each phosphoprotein. All data are presented as mean ± SE (*n* = 13 per bar). Beneath each bar graph, representative images of the magnitude of fold change are pictured. (F, G) The relationship between p‐p70s6k expression at 15mPOST and 90mPOST each condition and the percentage of fast‐twitch fibers. # indicates significant time effect whereby values were significantly less than baseline regardless of load condition (*P* < 0.05).

No significant effects of time (*F*_1,13_ = 0.40, *P* = 0.539) or load (*F*_1,13_ = 0.02, *P* = 0.901) occurred for p‐AMPK (Thr172) protein expression. A statistical trend for significance for a load × time interaction occurred (*F*_1,13_ = 3.50, *P* = 0.084), however, dependent samples *t*‐test revealed no significant differences between loads at POST or 90mPOST (Fig. 7A). No significant effects of time (*F*_1,13_ = 0.46, *P* = 0.511), load (*F*_1,13_ = 1.16, *P* = 0.301), or load × time interaction (*F*_1,13_ = 0.26, *P* = 0.621) occurred for p‐mTOR (Ser2448) protein expression (Fig. 7B). No significant effects of time (*F*_1,12_ = 0.99, *P* = 0.339) or load (*F*_1,12_ = 0.09, *P* = 0.773) occurred for p‐p70s6k (Thr389) (Fig. 7C). However, a trend for significance a load × time interaction occurred (*F*_1,12_ = 24.77, *P* = 0.056). Dependent samples *t*‐test revealed no significant differences in p‐p70s6k expression between conditions at any time point (*P* > 0.05). A significant main effect of time (*F*_1,13_ = 16.71, *P* = 0.001) occurred for p‐rps6 (Ser235/236) protein expression (Fig. 7D). However, no significant effect of load (*F*_1,13_ = 0.17, *P* = 0.689) or load × time interaction (*F*_1,13_ = 0.01, *P* = 0.912) occurred. A statistical trend for significance occurred for an effect of time regarding p‐4EBP1 (Thr37/46) protein expression (*F*_1,13_ = 3.74, *P* = 0.075). However, no significant effects of load (*F*_1,13_ = 2.51, *P* = 0.137) or load × time interaction (*F*_1,13_ = 2.22, *P* = 0.160) occurred (Fig. 7E). Moderate associations that trended toward significance existed between fast fiber percentage and p‐p70s6k expression at 15mPOST heavy RE (*r* = 0.50, *P* = 0.06; Fig. 7G) and 90mPOST light RE (*r* = 0.51, *P* = 0.06; Fig. 7F). Nonsignificant associations existed between fast fiber percentage and p‐p70s6k expression at 15mPOST light RE (*r* = 0.39, *P* = 0.15; Fig. 7F), and 90mPOST heavy RE (*r* = 0.37, *P* = 0.17; Fig. 7G).

## Discussion

Collectively, our data suggest similar molecular albeit distinct neuromuscular and functional recovery responses to light and heavy RE. The primary findings of this investigation can be summarized as follows: (1) although no significant difference in total volume load between conditions occurred, the total number of repetitions required to achieve concentric repetition failure and thus time required in the light condition was significantly greater, (2) EMG_AMP_ was significantly higher during heavy RE, while iEMG was significantly greater during light RE, and (3) functional leg extensor recovery was significantly dampened after light RE. These findings are described in greater detail below.

### Electromyography differences between light and heavy load lifting

The results of the present study indicated that EMG_AMP_ increased from the first to the last repetition (independent of set) and across sets in both the heavy and light RE conditions. However, EMG_AMP_ remained greater in the high‐load compared to low‐load RE condition across all sets. Despite achieving greater EMG_AMP_, the total volume of activation (i.e., iEMG) was greater during the low‐load RE condition. Therefore, the results indicated that although both high‐ and low‐load RE completed to volitional exhaustion necessitated an increase in muscle EMG_AMP_, EMG_AMP_ remained greater in the high‐load condition. However, the fatigue‐induced increase in EMG_AMP_ combined with the greater number of repetitions and time under load associated with low‐load RE resulted in a greater volume of activation during light than heavy RE. These results agree with and/or extend those of several previous studies (Akima and Saito 2013; Schoenfeld et al. 2014; Jenkins et al. 2015). For example, Schoenfeld et al. (2014) observed greater peak EMG_AMP_ values during a set of leg press exercise performed to failure using 75% 1RM compared to 30% 1RM. Similar to the present study, Jenkins et al. (2015) observed that leg extension RE to failure at both 80% and 30% 1RM resulted in an increase in activation from the first to the last repetition, but that EMG_AMP_ was consistently greater in the 80% condition. Jenkins et al. (2015) also reported that, despite achieving greater EMG_AMP_ during the 80% 1RM condition, total iEMG was greater in the 30% 1RM condition.

Although EMG_AMP_ is related to the net motor unit activity and increases monotonically with an increase in neural drive to the muscle (Farina et al. 2004), the surface EMG signal can also be affected by factors such as fiber membrane properties (such as motor unit conduction velocity and action potential shape), amplitude cancellation, etc. Thus, the degree to which greater EMG_AMP_ values during high‐load training may be interpreted as the result of greater neural drive to the muscle is limited (Jenkins et al. 2016b). Future studies utilizing more accurate techniques, such as EMG signal decomposition and/or spike triggered averaging or examination of muscle glycogen contents, are needed to more fully understand the similarities or differences in muscle activation during high‐ and low‐load resistance training.

### Functional recovery differences between light and heavy load lifting

Our operational definition of recovery is a reestablishment of the initial state, thereby distinct yet required for adaptation (e.g., improving select parameters above initial status)(Sands et al. 2016). Relative to PRE, recovery of peak torque at all isokinetic velocities at POST was significantly dampened after light RE compared to recovery from heavy RE. Additionally, the percent change in average peak torque statistically collapsed across velocities from baseline to 48hPOST was substantially lower after light RE. Given that the soreness and muscle damage responses after each condition were not significantly different (based on algometry measurements and serum myoglobin concentrations), other mechanisms seem responsible for this differential recovery response. To our knowledge, no studies have compared the acute (i.e., up to 48 h after) functional recovery kinetics of light and heavy RE to concentric repetition failure directly, within subjects. Thus, no direct comparisons can be made with other data. However, inferences can be made in light of available evidence pertaining to the recovery responses to light and heavy RE, and explanatory mechanisms of our neuromuscular data follow.

Etiological mechanisms of fatigue are generally divided into anatomical locations; namely, central (i.e., brain and spinal cord) and peripheral (i.e., structures outside of the brain and spinal cord) (Allen et al. 2008). In the present study, EMG_AMP_ was significantly greater during all sets of heavy RE. Therefore, since total volume load of RE was not significantly different between loads, it is plausible that the heavy condition would have exhibited a more impaired neuromuscular recovery response than light. Indeed, other investigations have shown more pronounced decrements in force production and recovery kinetics after heavy RE compared to moderate and light RE (Linnamo et al. 1998; Raastad and Hallen 2000; McCaulley et al. 2009). However, the aforementioned studies are limited in comparative validity to the present study as subjects did not train to failure and volume load was lower in the light conditions (Linnamo et al. 1998; Raastad and Hallen 2000). While speculative at best, we posit that low‐frequency fatigue related to skeletal muscle calcium release and/or sensitivity occurred to a greater degree following the low‐load condition. Low‐frequency fatigue has been described as a long‐lasting loss in the mechanical response of skeletal muscle following exercise that is independent of changes in action potential morphology or the contractile mechanism itself, and low‐frequency fatigue manifests at low stimulation frequencies (Chin and Allen 1996; Ratkevicius et al. 1998). Although the exact mechanism(s) for low‐frequency fatigue have not been clearly elucidated, it is thought to be caused by a calcium (Ca^2+^) activated process and is more pronounced when greater concentrations of Ca^2+^ exist in the sarcoplasm during maintained or prolonged contractions (Chin and Allen 1996; Ratkevicius et al. 1998). Furthermore, Chin and Allen (1997) suggest that reductions in Ca^2+^ release and force production following fatiguing contractions are partially explained by decreased muscle glycogen concentrations. Therefore, given the much greater time under load and greater total volume of muscle activation during light RE, it is conceivable that light RE resulted in greater low‐frequency fatigue than did heavy RE and caused a greater impairment in force production that persisted up to 48 h after exercise. Evidence suggests that low‐frequency fatigue requires several days to recover from and may be greater after longer duration, repetitive exercise compared to exercise of shorter durations (Edwards et al. 1977; Bigland‐Ritchie et al. 1986; Bystrom and Kilbom 1991; Ratkevicius et al. 1998). Thus, light load training may propagate low‐frequency fatigue compared to heavy load training and this may explain why recovery torque was impaired in the former condition up to 48 h postexercise. Notably, we did not assay indices related to low‐frequency fatigue (e.g., muscle glycogen depletion or Ca^2+^ release kinetics), so our speculation is limited in this regard. Notwithstanding, the impaired functional recovery up to 48 h following light RE has practical implications for the trainee.

### Intramuscular–molecular comparisons between light and heavy load lifting

An impactful series of recent studies from Stuart Phillips' group with a similar focus as the present study have demonstrated the following: (1) light (30% 1RM), unilateral knee extension RE to failure resulted in significantly greater myofibrillar fractional protein synthesis rates than heavy RE to failure (90% 1RM) 24 h after RE (Burd et al. 2010), (2) significant and statistically equal increases in quadriceps muscle volume after 10 weeks of unilateral knee extension RE three times per week with either 30% 1RM for three sets to failure or 80% 1RM for three sets to failure occurred, while greater improvements in strength occurred in the heavy group (Mitchell et al. 2012), and (3) significant and statistically equal increases in lean body mass and muscle fiber cross‐sectional area occurred from both light (30–50% 1RM) and heavy (75–90% 1RM) full‐body RE to failure after 12 weeks (Morton et al. 2016b). Moreover, other researchers have demonstrated that lighter (50–65% 1RM) and heavier RE (80–85% 1RM) elicit similar postexercise profiles in myosin heavy chain mRNA responses (Schwarz et al. 2015), and phosphoprotein signaling responses related to MAPK and mTORC1 signaling (Taylor et al. 2012). Herein, we contribute to these molecular findings by assaying a series of mRNAs that have been implicated in the adaptive response to exercise and report that significant main effects of time occurred for MuRF‐1, MSTN, and PGC1‐*α*. Specifically, MuRF‐1 mRNA increased approximately 400% by 90mPOST RE in both conditions. MuRF‐1 is an E3 ligase responsive to exercise that is involved in proteolytic/atrophic degradation of proteins involved in the ubiquitin/proteolysis pathway (Louis et al. 2007). Our data agree with Louis et al. (2007) who reported an approximate fourfold increase in MuRF‐1 mRNA expression 1 h after RE. Based on these data, the increase in MuRF‐1 mRNA expression after RE seems to occur whether light or heavy loads are employed. MSTN is a negative regulator of muscle hypertrophy, and tends to be highly expressed in atrophic conditions (Jones et al. 2004; Kim et al. 2005; Louis et al. 2007). Although not to the same extent of downregulation as reported by Louis et al. at 1 h after RE (~300% downregulation) (Louis et al. 2007), MSTN mRNA expression exhibited a significant time effect whereby at 90mPOST, levels were ~30% lower compared to PRE levels. While it is difficult to discern as to whether or not this finding is meaningful, it is notable that MSTN mRNA and protein levels have also been reported to be downregulated in response to chronic resistance training (Kim et al. 2007; Hulmi et al. 2009). Hence, an acute downregulation in MSTN mRNA expression following light and heavy RE could be related to increases in muscle hypertrophy reported in longer term studies implementing both training loads. PGC1‐*α*, although involved in many coordinated signaling networks, has been implicated as a key promoter of metabolic gene expression and, specifically, mitochondrial biogenesis (Wu et al. 1999; Lehman et al. 2000). PGC1‐*α* mRNA expression increased from approximately two‐ to fourfold after both bouts of RE. This extent of upregulation after exercise agrees with other literature (Norrbom et al. 2004; Mathai et al. 2008), and suggests that a significant increase in PGC1‐*α* gene expression 90 min after RE occurs to largely the same extent regardless of the magnitude of load.

Similar to mRNA expression responses, phosphoprotein expression alterations after each bout of RE were similar between load conditions, and only significant main effects of time were observed for p‐4EBP1 and p‐rps6. Significant decreases in p‐4EBP1 at both 15mPOST and 90mPOST were observed, while significant increases in p‐rps6 occurred at 15mPOST but decreased back to baseline levels by 90mPOST. While fold changes in p‐AMPK, p‐mTOR, and p‐p70s6k at 15mPOST and 90mPOST were not significant herein, p‐AMPK and p‐p70s6k expression load × time interaction effects approached our a priori alpha level of significance (*P* = 0.08, *P* = 0.06) and, consequently, we consider these trends meaningful in the sense of a true percentage‐based interpretation.

p‐AMPK was not measured in the aforementioned studies to which our protein and mRNA expression data are primarily being compared, but was deemed important to include in this investigation given its role in the adaptive response to RE. p‐AMPK has been shown to transiently attenuate mTORC1 pathway activation and thereby reduce the initiation of protein synthesis by activating TSC2 in response to the accumulation of AMP caused by the catabolism of ATP during exercise (Inoki et al. 2003; Thomson et al. 2008). p‐AMPK increased ~200% after heavy RE and decreased slightly by 90mPOST. Interestingly, no clear increase in p‐AMPK was observed 15mPOST light RE but increased by ~300% at 90mPOST relative to baseline. Thus, it seems that p‐AMPK expression exhibits a delayed response after light RE compared to heavy RE, although this effect did not reach significance in the present study. Wang et al. (2011) showed similar increases in p‐AMPK expression 1 h after RE with loads ranging from 70% to 80% 1RM, although subjects also completed cycling exercise. Interestingly, Popov et al. (2015) reported a similar trend in p‐AMPK after RE where expression values were significantly higher 45 min after relatively higher intensity RE (~75% 1RM) compared to a reduced expression after lower intensity RE (~50%1RM) at the same time point. Thus, these data suggest a more pronounced expression of p‐AMPK after heavy RE at ~1 h after RE while a slightly delayed, but eventual increase seems to occur after light RE (Popov et al. 2015).

mTOR is a protein kinase convergence point of many anabolic signals in muscle tissue. Upon phosphorylation at Ser2448, mTOR can initiate protein synthesis via the hyperphosphorylation and inhibition of 4EBP1 and/or phosphorylation of p70s6k at Thr389 (Baar and Esser 1999; Bodine et al. 2001; Ogasawara et al. 2016). We did not observe a significant increase in p‐mTOR expression 1 h POST either bout as reported by Mitchell et al. (2012), but rather a quantitative downregulation at both POST and 90mPOST. Dreyer et al. (2006) also reported significant increases in p‐mTOR expression 1 h after RE. However, Wilkinson et al. (2008) reported no significant increase in p‐mTOR expression immediately and 4 h after three sets of ten repetitions with 80% 1RM. Additionally, Creer et al. (2005) did not report a significant increase in p‐mTOR expression immediately after and up to 10 h after RE. Camera et al. investigated the early time course of the mTOR signaling pathway after RE and reported no significant increase in p‐mTOR until 30 min after RE and a decrease in p‐mTOR expression by 1 h after RE toward basal levels of expression. While it is difficult to reconcile why these data collectively report differential mTOR phosphorylation patterns in response to resistance exercise, we speculate that it could be due to differences in study design (e.g., training style implemented, fed state of subjects, biopsy sampling time points). Of the aforementioned studies, only our study and that of Mitchell et al. trained participants to volitional fatigue. Hence, differences in findings between our work and Mitchell et al. versus other reports may be due to altered phosphorylation states of mTORC1 signaling intermediaries when training to volitional fatigue, which provides direction for future work. With regard to feeding differences between studies, it is notable that Mitchell et al. also fed participants a source of high‐quality protein (i.e., a protein bar containing 360 kcal, 3.5 g leucine, 30 g protein), whereas our participants received no nutritional provision following exercise. Hence, their report of postexercise increases in mTORC1 markers may be due to the combination of exercise and nutrient‐stimulated mTOR phosphorylation.

p‐4EBP1 expression was reduced at both 15mPOST and 90mPOST RE. 4EBP1 is a protein that binds to eukaryotic initiation factor 4E preventing translation initiation of mRNA and thereby protein synthesis to occur, but, when phosphorylated at Thr37/46, allows translation initiation to proceed (Tsai et al. 2015). Given the quantitative downregulation of p‐mTOR at Ser2448, this finding is sensible as p‐mTOR phosphorylates 4EBP1 to allow eventual translation initiation. Burd et al. (2010) reported significant increases in p‐4EBP1 expression at 4 h after both light and heavy RE. The time at which muscle tissue was collected likely explains the difference in findings, as Dreyer et al. (2006) have reported similar reductions in p‐4EBP1 expression for up to 2 h after 100 repetitions of leg extensions ranging from 60% to 70% 1RM. Thus, these data suggest a transient decrease in p‐4EBP1 15mPOST and 90mPOST RE to failure regardless of load magnitude.

p‐p70s6k, although quantitatively greater at POST and 90mPOST, did not reach significance at either time point after either condition. However, p‐p70s6k expression produced the largest increases in magnitude of fold change where means at each time point after RE revealed five‐ to sevenfold increases. Burd et al. (2010) reported ~50% increases in p‐p70s6k expression 4 h after light RE only, with no significant change after heavy RE. While our findings somewhat disagree with Mitchell et al. (2012) who also compared light and heavy RE p‐p70s6k expression immediately post and 1 h post RE and reported significant increases above baseline at both time points, the expression patterns from this investigation qualitatively resemble these authors' findings.

rps6 is a ribosomal protein that is a component of the 40s ribosomal subunit and is involved in the regulation of the translation of mRNA, and therefore protein synthesis (Meyuhas 2008). Upon phosphorylation by p70s6k, rps6 allows protein synthesis to proceed (Bolster et al. 2003). Phosphorylated rps6 was not analyzed in any of the aforementioned investigations comparing light and heavy RE. However, data from Bolster et al. (2003) explicitly agree with our findings showing the highest increases in p‐rps6 15 min following RE and a decrease toward basal levels thereafter.

Collectively, our mRNA and phosphoprotein data add to the current body of literature comparing light versus heavy leg extensor training to failure and, while there are numerical trends between conditions, all of the assayed markers elicited similar postexercise responses. Notably, since several potentially relevant metabolite variables were not measured, the similar responses in our molecular analyses could have been due to light and heavy RE eliciting similar increases in metabolite accumulation (e.g., lactate, inorganic phosphate), substrate depletion (e.g., intramuscular glycogen and phosphocreatine), and/or mechanotransduction responses consequently leading to similar molecular responses. In this regard, future studies implementing similar study designs could continue to explore these possibilities.

### Experimental considerations

Unresolved limitations in our current study should be noted which may have affected measured variables. These include: (1) the possible influences of repeated biopsies, (2) the possible influence of postloading tests contributing to the molecular responses, (3) a possible confounding effect of a novel stimulus in the light RE condition for most subjects that likely habitually train in the 8–12 repetition range, and (4) a lack of nervous system analytical techniques to determine the site of fatigue (i.e., central or peripheral). Additionally, while we feel a strength of our study lies in the within‐subject crossover design, our study is limited in the fact that we lacked appropriate experimental power to detect small, main effects of training on certain molecular variables. In this regard, we observed robust (albeit nonsignificant) postexercise increases in the phosphorylation levels of p70s6k, AMPK, and rps6 as well as the mRNA expression of TNF‐*α* and IL‐6. Hence, we posit that future studies continuing to pursue related research questions likely need to examine the training responses in greater than 15 subjects to detect small, yet meaningful, significant effects of load.

It should also be noted that a notable difference between the current study and that of Burd et al. (2010) to which we compare several of our findings is the implementation of 80% 1RM loads herein and 90% 1RM loads by Burd et al. Indeed, Kumar et al. (2009) report that leg extensor training at 75% 1RM (3 sets of 8 repetitions) and 90% 1RM (6 sets of 3 repetitions) elicit similar postexercise myofibrillar protein synthetic responses in younger men. Hence, this finding supports the contention that acute molecular markers should have responded similarly between our subjects versus the subjects examined by Burd et al. However, a review by Fry (2004) examining over 16 RT studies suggests that myofiber hypertrophy increases as a function of training intensity when 40–95% 1RM loads are implemented. Alternatively stated, differences between our data and the data reported by Burd et al. could be due to the implementation of a 10% greater relative lifting intensity in their study which could have seemingly elicited greater muscle anabolism.

Moving forward, we believe that certain experimental considerations should be noted related to the currently allocated study design. First, none of the studies comparing light versus heavy load lifting (including the current study) have assayed direct markers of muscle proteolysis, and it is notable that the intramuscular response to a single bout of RE does not necessarily represent the long‐term adaptive response, particularly when measuring markers of muscle protein synthesis without muscle protein breakdown (Murton and Greenhaff 2013; Camera et al. 2016). Second, evidence indicates that periodized RT (i.e., logical, sequential manipulation of training variables) is more effective than nonperiodized RT (Fleck 1999; Rhea and Alderman 2004). Thus, training to failure may be suboptimal for long‐term improvements in strength and hypertrophy (Stone et al. 1996; Peterson et al. 2004; Izquierdo et al. 2006). Finally, protein synthesis, as well as mRNA and protein expression alterations, measured via a ~150 mg muscle biopsy sample are unlikely to mirror alterations in other muscles in context of full‐body RT (Murton and Greenhaff 2013; Camera et al. 2016). Given that all of the above‐mentioned studies (including our own) have used leg extensor training in order to address whether light versus heavy load lifting is comparatively more effective, future research is needed in order to determine if this applies to compound movement exercises aimed at increasing full body muscle hypertrophy and strength.

## Conclusions

Largely in accordance with our hypothesis, we demonstrate that, beyond select markers of prolonged fatigue occurring following light load RE, the molecular responses to light and heavy RE are similar. As stated earlier, unresolved questions remain regarding how light and heavy RE affect metabolite accumulation, substrate depletion, and/or mechanotransduction responses. Hence, future work in this area can elucidate how these different training modalities affect these processes using valid, reliable analytical techniques for more direct predictive relationships.

## Conflict of Interest

The authors have no conflicts of interest to disclose.
